# Autoimmune thyroiditis as a risk factor for stroke: A historical cohort study

**DOI:** 10.1212/WNL.0000000000000377

**Published:** 2014-05-06

**Authors:** André Karch, Sara L. Thomas

**Affiliations:** From the Department of Epidemiology (A.K.), Helmholtz Centre for Infection Research, Braunschweig, Germany; Faculty of Epidemiology & Population Health (A.K., S.L.T.), London School of Hygiene and Tropical Medicine, UK; and Department of Neurology (A.K.), University Medical Center Göttingen, Germany.

## Abstract

**Objective:**

To investigate the effect of autoimmune thyroiditis (AIT) on risk of stroke and to assess whether any increased risk (1) varied by AIT duration, and (2) was independent of classic cardiovascular risk factors.

**Methods:**

This was a large historical cohort study using data from The Health Improvement Network Database. Rates of first stroke during follow-up in thyroxine-treated patients with AIT (n = 34,907) were compared with those in matched individuals without AIT (n = 149,632) using random-effects Poisson regression models.

**Results:**

There was strong evidence for a slightly increased risk of stroke in patients with AIT (adjusted rate ratio = 1.10, 95% confidence interval: 1.01–1.20). The observed increase was partly independent of cardiovascular risk factors. Higher effect sizes were identified in the first year after AIT diagnosis (rate ratio = 1.33, 95% confidence interval: 1.14–1.56) but not in the long-term, consistent with a residual effect of hypothyroidism.

**Conclusion:**

Our results support the hypothesis of a slightly increased risk of stroke in patients with AIT. The higher effect size found soon after AIT diagnosis suggests an increased cardiovascular risk due to thyroid-hormone deficiency rather than a cumulative effect of autoimmune pathology. Better screening and early treatment of patients with asymptomatic hypothyroid AIT could help reduce excess risk of stroke in the first year after diagnosis.

Autoimmune thyroiditis (AIT) is one of the most common autoimmune diseases in Western countries.^1,2^ In iodine-deplete areas, such as the United Kingdom, AIT causes >90% of all noniatrogenic cases of hypothyroidism, and the terms are often used interchangeably.^3,4^

Individuals with hypothyroidism/AIT have long been hypothesized to be at increased risk of cardiovascular events, with a recent meta-analysis suggesting an increased risk of approximately 20% for coronary heart disease (CHD).^5–14^ Potential causal pathways between hypothyroidism and CHD include hyperlipidemia, hypertension, diabetes, and obesity.^3,15–21^

Unlike CHD, there have been few epidemiologic studies of whether hypothyroidism increases cerebrovascular disease risk. These have reported conflicting results and were mostly small studies with methodologic limitations.^7,21–26^ Cerebrovascular risk factors (e.g., increased carotid artery intima-media thickness and atrial fibrillation [AF]) have been linked to AIT or hypothyroidism of unspecified cause, suggesting pathways from AIT to stroke independent of general cardiovascular risk factors.^23,27^ Furthermore, autoimmune pathology in AIT might itself affect cerebrovascular risk, as has been shown for other autoimmune diseases.^16,28^

These uncertainties have led to calls for large cohort studies to investigate the association between AIT and cerebrovascular disease.^21^ The primary objective of our study was to assess the effect of AIT on risk of stroke and TIA using a large dataset derived from UK primary care records. Secondary objectives were to examine to what extent any effect of AIT on cerebrovascular disease (1) was independent of classic cardiovascular risk factors, and (2) could be attributable to thyroid hormone deficiency or autoimmune pathology.

## METHODS

### Study design and study population.

This was a historical cohort study using data from The Health Improvement Network (THIN) database. THIN is a population-based database of electronic health records of approximately 6 million patients from more than 300 general practices in the United Kingdom.^29^ THIN has been validated for a wide range of medical conditions, including stroke, and individuals contributing data to THIN are representative of the UK population.^30,31^ Data available include prescribed medications, medical diagnoses, lifestyle conditions, demographic/personal information, and feedback from specialist appointments and hospital admissions. The present study population was a subset of a larger THIN study, in which patients with an autoimmune disease enrolled in THIN between 1987 and 2007 had each been matched by age, sex, and general practice to up to 6 individuals without any autoimmune disease.

### Exposure and outcome.

AIT was the exposure of interest; unexposed individuals comprised patients without AIT (or any other autoimmune disease) who had been matched on age, sex, and general practice to the patients with AIT in the original THIN dataset. The outcome of interest was first-ever stroke (including ischemic and hemorrhagic) or TIA during follow-up. Exposure and outcome were defined using prespecified Read code lists (appendix e-1 on the *Neurology*® Web site at Neurology.org).

### Study period.

Start of follow-up for individuals with AIT and their unexposed counterparts was the date of the first thyroxine prescription in the patients with AIT (the index date). End of follow-up was defined as the day of the outcome (stroke or TIA) or end of follow-up in THIN (death, transfer out, or the practice's last data-collection date).

### Eligibility criteria.

**Figure 1:**
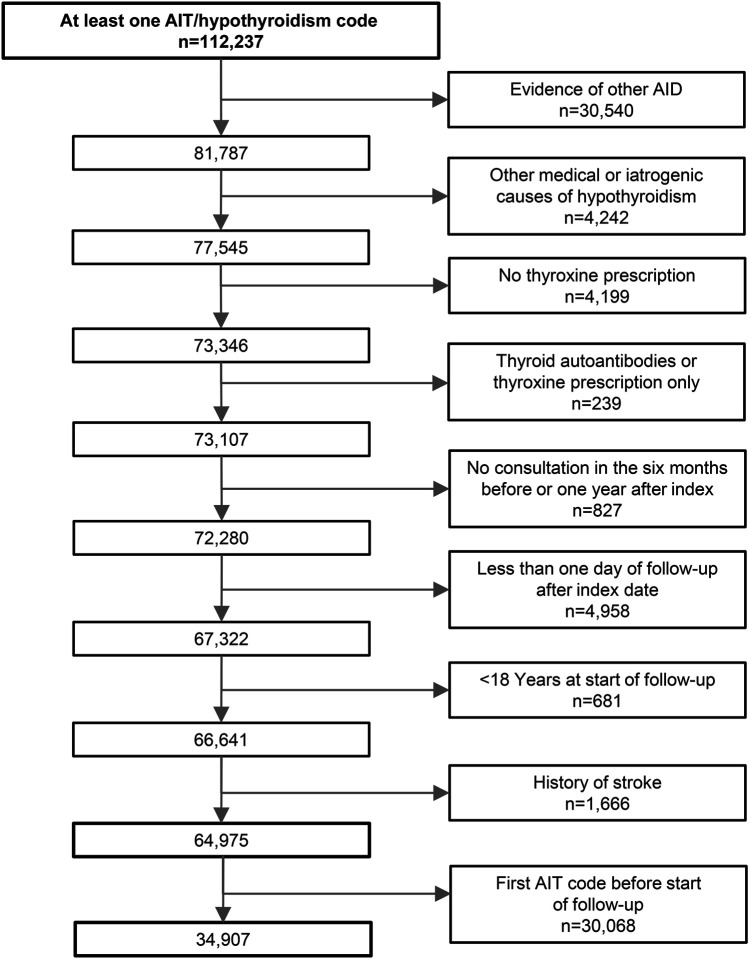
Flowchart of the selection process for patients with AIT AID = autoimmune disease; AIT = autoimmune thyroiditis.

Patients with AIT must have been first diagnosed with AIT during active follow-up in THIN and have been prescribed thyroxine during follow-up. Individuals with other causes for hypothyroidism (e.g., previous thyroidectomy) were excluded from analyses (figure 1). Diagnoses for past or ongoing conditions (including AIT) are sometimes recorded retrospectively in the first few months after a patient registers with a practice.^30^ To ensure that we enrolled incident cases, we excluded patients with AIT who were diagnosed during their first year of follow-up in THIN. All individuals (exposed and unexposed) who had a code for stroke or TIA before start of follow-up, or who were younger than 18 years, were not considered for inclusion. Also, because the unexposed (non-AIT) individuals had no other autoimmune disorders, individuals with AIT who had a preexisting or who developed a second autoimmune disorder were also excluded. Unexposed individuals had to be actively enrolled in THIN at the index date of their matched individual with AIT; to ensure that they were truly active, they had to have a consultation with the practice in the 6 months before or in the year after the index date of the patient with AIT.

### Analysis.

**Figure 2:**
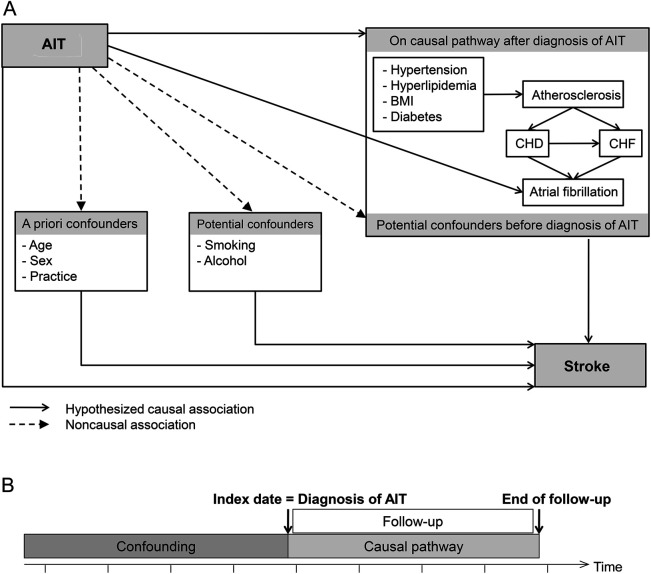
Hypothesized interrelationships among AIT, stroke, and other variables (A) Conceptual framework for association between AIT and stroke. (B) Differentiation between confounding and mediating effects of factors hypothesized to be on the causal pathway. Presence of factors at the index date was assumed to be attributable to confounding in the primary analysis. AIT = autoimmune thyroiditis; BMI = body mass index; CHD = coronary heart disease; CHF = congestive heart failure.

In the conceptual framework for this study (figure 2), cofactors were categorized as follows: (1) a priori confounders (sex, current age, general practice); (2) other potential confounders (smoking, alcohol consumption, calendar year, time in study); and (3) factors that could be either confounders or on the causal pathway from AIT to stroke, depending on whether they occurred before or after AIT diagnosis (figure 2B). Missing data for smoking, alcohol, and body mass index (BMI) were treated using the missing indicator method. An individually matched analysis is not needed in matched cohort studies, but inclusion of the matched variables in a multivariable analysis is advisable^32^; this also addressed any imbalances between exposed and unexposed patients regarding these variables after the application of exclusion criteria.

Statistical analyses were conducted using Stata 11 (StataCorp, College Station, TX). Baseline characteristics were compared using cross-tabulation, means or medians as appropriate, and χ^2^ tests, 2-sided *t* tests, and Wilcoxon rank-sum tests.

Main analyses were performed using random-effects multivariable Poisson regression models allowing for clustering within general practice. Four different models were built. The first adjusted for sex and current age. In the second model, we assessed potential confounding by smoking, alcohol consumption, calendar year, and time in study, introducing these variables sequentially into the model and retaining them if they changed the effect estimate of AIT on stroke incidence appreciably. The third model additionally assessed risk factors (hypertension, AF, hyperlipidemia, diabetes, CHD, congestive heart failure [CHF], and BMI) that were present at the index date and could therefore also confound the association between AIT and stroke. In the final model, we examined time-updated values of these factors for individuals who developed these conditions during follow-up to assess potential causal pathways between diagnosed AIT and stroke. We applied this analysis strategy to 2 main outcomes, first stroke, then stroke or TIA. In all models, standard errors were examined for evidence of colinearity, and *p* values were obtained using likelihood ratio tests.

We investigated effect modification by AIT duration as a proxy for the effects of hypothyroidism (likely to be evident in the early stages of disease, before adequate thyroxine replacement) vs the possible cumulative effect of autoimmunity (long-term increased risk, likely to occur later during follow-up). We also investigated effect modification by age (<60, 60–80, >80 years) and performed several sensitivity analyses. First, analyses were repeated starting follow-up at the date of first AIT code instead of first thyroxine script. Second, cardiovascular conditions present among patients with AIT at diagnosis could be early mediating factors for stroke rather than a confounding variable; to assess their importance, we performed subgroup analyses for individuals without a history of any cardiovascular disease–related conditions at baseline.

### Standard protocol approvals, registrations, and patient consents.

Ethics approval was obtained from the South-East Multicentre Research Ethics Committee and from the London School of Hygiene and Tropical Medicine Ethics Committee.

## RESULTS

In total, 184,539 eligible individuals from 287 general practices were included in this study, of whom 34,907 had AIT (figure 2) and 149,632 had no evidence of AIT. Individuals with AIT were slightly older than individuals without AIT (median 59.8 vs 57.0 years), were followed up for longer (median 3.2 vs 3.0 years), and a slightly higher proportion was female (81.5% vs 80.0%; table 1). Current smoking and heavy drinking were less common among those with AIT. For cardiovascular risk factors, individuals with AIT were more likely to have been diagnosed with hypertension, diabetes, hyperlipidemia, CHD, or CHF at baseline (all *p* < 0.001). This pattern remained after adjusting for the slight age and sex imbalances (data not shown).

**Table 1:**
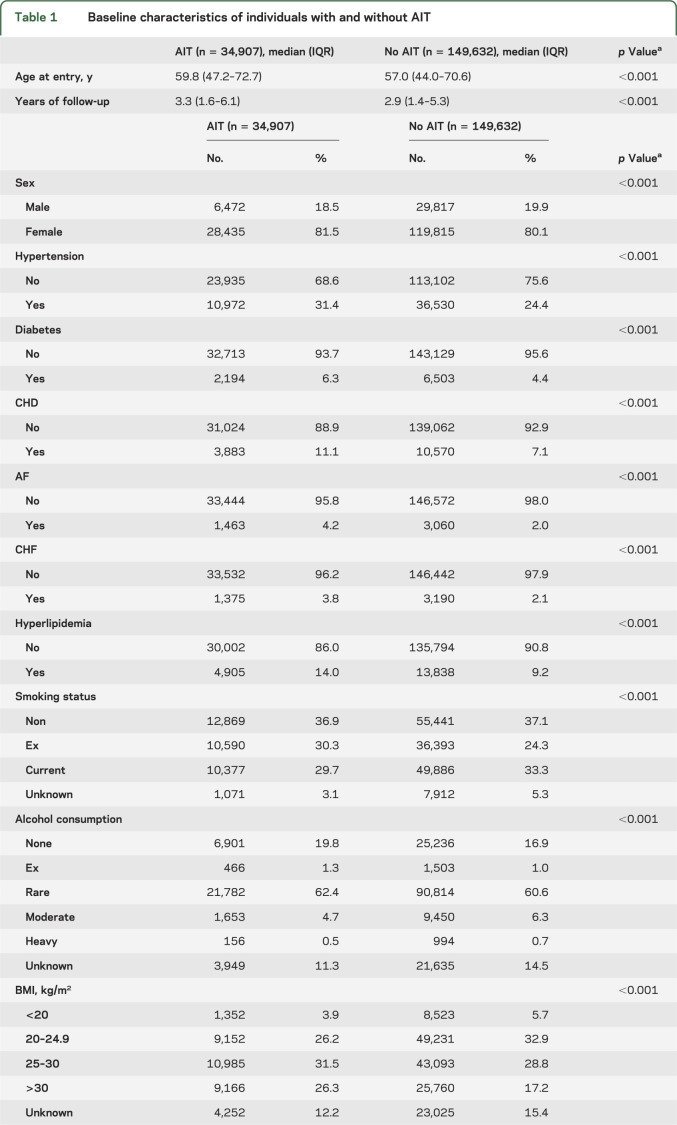

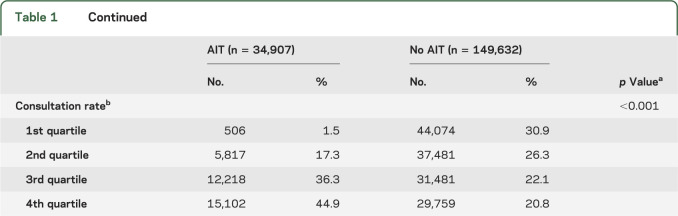
Baseline characteristics of individuals with and without AIT

Individuals with AIT had a 13% increased rate of stroke compared with individuals without AIT after adjusting for current age and sex and allowing for clustering in practice (model 1, table 2). The increased risk was very similar (rate ratio [RR] = 1.14, 95% confidence interval [CI]: 1.04–1.24, *p* = 0.003) after adjusting for alcohol and smoking (model 2) and was not affected by the duration of follow-up or calendar year. The RR decreased slightly to 1.10 (95% CI: 1.01–1.20, model 3) after adjusting additionally for cardiovascular risk factors present at baseline (hypertension, hyperlipidemia, AF, and BMI) and was not further changed by consideration of existing diabetes, CHD, and CHF.

**Table 2:**
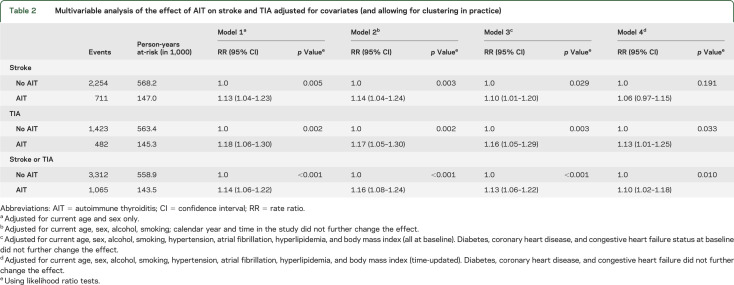
Multivariable analysis of the effect of AIT on stroke and TIA adjusted for covariates (and allowing for clustering in practice)

Individuals with AIT had an increased risk of developing CHD, hyperlipidemia, CHF, and diabetes during follow-up compared with unexposed individuals (table e-1), but not hypertension or AF. After adjusting for these potential mediating factors between AIT and stroke, the RR for stroke was further reduced to 1.06 (95% CI: 0.97–1.15, model 4). Effect sizes were slightly higher for all models when the outcome definition was expanded to both stroke and TIA (table 2).

**Figure 3:**
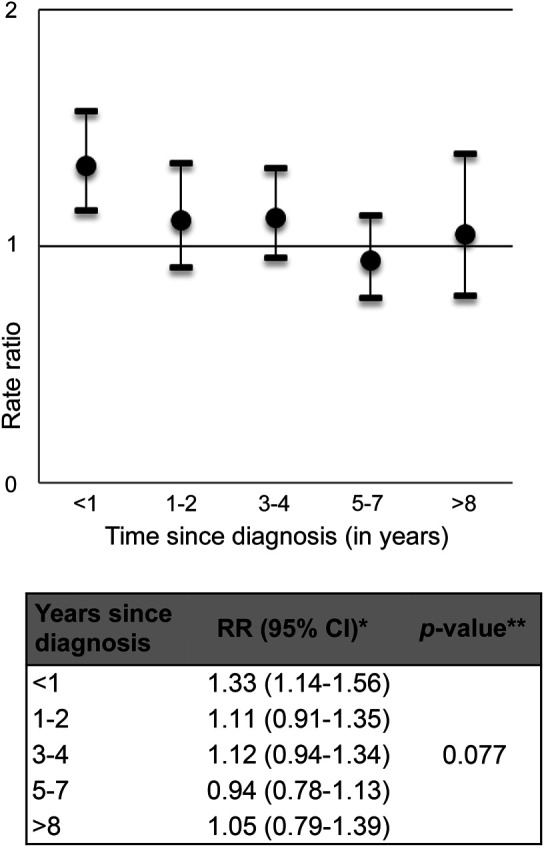
Effect of autoimmune thyroiditis on stroke stratified by time since diagnosis (first thyroxine script) *Adjusted for current age, sex, alcohol, smoking, hypertension, atrial fibrillation, hyperlipidemia, and body mass index (all at baseline) and allowing for clustering in practice. **Using a likelihood ratio test to compare a model with against a model without an interaction term for time since diagnosis. CI = confidence interval; RR = rate ratio.

There was evidence that the effect of AIT on stroke varied with duration of disease (*p*_interaction_ = 0.078, figure 3). The effect of AIT on stroke was largest in the first year after diagnosis (RR = 1.33, 95% CI: 1.14–1.56) and was increased both in the first 6 months (RR = 1.44, 95% CI: 1.17–1.78) and 7 to 12 months after start of treatment (RR = 1.22, 95% CI: 1.02–1.55). Subsequent RRs were stable with all 95% CIs including 1.0. Similar results were obtained for analyses using TIA or stroke and TIA alone as the outcome. There was no evidence that the effect of AIT varied with age (*p*_interaction_ = 0.516).

All sensitivity analyses showed results compatible to those obtained from the main analyses (data not shown).

## DISCUSSION

Our study provides strong evidence for a slightly increased risk of stroke in patients with AIT, particularly in the first year after AIT diagnosis. Our analyses indicated that people with AIT were more likely to develop hyperlipidemia, CHD, and CHF, adding evidence to previous discussions about whether AIT is associated with these factors, and that some, but not all, of the increased risk of stroke among patients with AIT was mediated via these classic cardiovascular risk factors.^14^

Few previous studies have focused on potential associations between hypothyroidism or AIT and stroke. Our systematic review identified 7 studies, which showed effect sizes ranging from 0.8 to 1.6 (table e-2). All of these studies except for the Scottish study from 2006 were small and had effect estimates with wide CIs that overlapped with those from the present study. Other methodologic limitations of previous studies included overadjustment for variables that could be mediators of increased stroke risk, and residual confounding (for example, the Scottish study lacked information on smoking, alcohol use, and medication history).^7,12,22–26^ Also, studies included individuals with a history of stroke. This could explain their slightly higher effect estimates, because thyroid hormone status is often assessed at stroke units, resulting in better AIT ascertainment in individuals with a stroke history (who are at higher risk of a subsequent stroke). Three of the studies were conceptually different from this study because they used asymptomatic patients identified as hypothyroid by laboratory tests who were mostly untreated with thyroxine.^7,12,25^

We found differences between individuals with and without AIT in the prevalence of traditional cardiovascular risk factors at baseline. Individuals with AIT were more likely to have AF and hypertension at baseline compared to those without AIT; however, they were not at increased risk of developing these conditions during follow-up. This finding is consistent with that from a recent population-based study from Germany, which reported that hypothyroidism was associated with prevalent but not incident hypertension.^33^ Hypertension and AF may not be on the causal pathway between AIT and stroke, although the differences we observed at baseline could represent early changes induced by undiagnosed AIT. Differences in AF (and possibly hypertension) at baseline between individuals with AIT and those without could also be explained by differences in ascertainment of AIT, if individuals with AF were more likely to have thyroid-stimulating hormone levels measured.

Our main model of interest, adjusted for these baseline variables, is based on the assumption that pathologic mechanisms on the causal pathway to stroke did not start until after AIT diagnosis and initiation of thyroxine treatment. Thus, these factors were treated as potential confounders. However, it is likely that the date of AIT diagnosis/start of treatment did not accurately capture start of disease. Given that patients with AIT often present with nonspecific symptoms, diagnosis of AIT can be delayed. Thus, it is plausible that cardiovascular causal processes could have started before formal diagnosis of AIT. If so, model 3 (which adjusted for these variables) may have provided a conservative estimate of relative stroke risk, and model 2 might provide an estimate closer to the true effect. However, the effect estimates produced by these 2 models were similar; also, the sensitivity analysis excluding all individuals with cardiovascular disease–related conditions at baseline showed results similar to the main analysis.

Our finding of a higher effect of AIT on stroke risk in the first year after diagnosis is compatible with the hypothesis of increased cerebrovascular risk in patients with AIT due to prediagnosis hypothyroidism and the time taken to reduce this risk after thyroid hormone replacement. There was no evidence for an increasing effect with increased length of follow-up (a possible proxy for a long-term effect mediated by autoimmune processes). An alternative explanation is that the observed effect of AIT on stroke was caused by thyroxine treatment because thyroxine can have a procoagulant effect.^34^ However, if the observed effect was due to thyroxine, we might expect to have seen the excess stroke risk restricted to the period soon after start of treatment, whereas the risk remained increased 6 to 12 months after diagnosis in our study. Moreover, a thyroxine-mediated effect might also be expected to generate short-term differences in incidence of AF between patients with AIT and individuals without AIT because thyroxine in high doses can induce AF; this was not the case. Nevertheless, we cannot exclude that some of the effect attributed to hypothyroidism could have been due to thyroxine, and we were unable to examine this formally because all patients with AIT received thyroxine.

This study has several strengths. We used a considerably larger study population than all previous studies, and thus had greater power to ascertain effect estimates. We had information on a variety of potential confounding factors that could be considered in the analysis. Unlike previous studies, we excluded individuals with a history of stroke, avoiding introduction of bias. Moreover, excluding people with other autoimmune diseases from our study population enabled us to observe the effect of AIT unmodified by other autoimmune disorders. This might not have been the case in other studies, because patients with AIT are at higher risk of acquiring other autoimmune disorders, some of which (e.g., rheumatoid arthritis, type 1 diabetes) are associated with considerably increased risk of cardio- and cerebrovascular disease.^16,35^

Potential limitations of our study also need consideration. Almost all noniatrogenic hypothyroidism in the United Kingdom is due to AIT, and we excluded individuals who had other reasons for hypothyroidism. However, our Read code list included nonspecific hypothyroidism codes, which increased sensitivity of AIT diagnosis but could have included a few non-AIT cases. Because hypothyroidism can remain undiagnosed for an unspecified time, some patients with AIT could have been misclassified as unexposed. This is likely to have happened largely independently of subsequent stroke risk, and thus may have driven the RR toward 1.0. Those with preexisting AF could have been more likely to have their AIT diagnosed, which could have resulted in some overestimation of the effect size of AIT on the risk of stroke. However, our sensitivity analyses excluding individuals with AF at baseline indicate that this is unlikely to have been a major problem.

Differential misclassification of the outcome was unlikely in this study because stroke is a serious condition and should have been recorded equally in both groups independent of AIT status, or health-seeking behavior. However, we could not differentiate between cases of ischemic and hemorrhagic stroke, because 85% of stroke codes did not specify the stroke subtype, consistent with previous studies of stroke using electronic health data.^23,36^ We hypothesized that AIT might be associated specifically with ischemic stroke because hypothyroidism is linked with classic risk factors for ischemia.^3,15–18,20,21,37^ Moreover, carotid artery intima-media thickness and AF, 2 large risk factors for ischemic stroke, have been linked to AIT.^22,23,27^ Ischemic stroke causes approximately 80% of stroke cases in adults, and effect sizes for TIA (an ischemic process) were comparable to those shown for stroke in our study, further supporting that AIT is associated with ischemic stroke. Nevertheless, AIT could also affect the risk of hemorrhagic stroke, because hypothyroidism has been shown to be associated with the development of von Willebrand syndrome and bleeding events.^38^

Individuals with AIT had higher consultation rates than those without AIT. It is therefore possible that they were more likely to be diagnosed and treated for conditions such as hyperlipidemia or hypertension. Monitoring and prompt treatment of these conditions could have decreased stroke risk associated with these conditions and might partly explain the relatively small increase in stroke risk associated with AIT. Nevertheless, this study provides a realistic picture of the residual effect of AIT after standard treatment and surveillance in the United Kingdom. Data concerning the potential confounders of ethnicity and socioeconomic status were not available. However, socioeconomic status was partly accounted for by allowing for clustering by practice.

Although THIN has been shown to be representative for the UK population, the results of this study might not be generalizable to all patients with AIT. We included only patients with AIT who received thyroxine treatment. This excluded patients who had never had hypothyroidism. If hypothyroidism is on the causal pathway between AIT and stroke, the observed effect relates to treated rather than all patients with AIT. Excluding individuals with AIT who had a second autoimmune disorder allowed us to pinpoint the specific effect of AIT, but may have excluded some patients with AIT who had stronger autoimmune processes and thus might not have captured all of the long-term risk of stroke mediated by autoimmune pathology.

Our study has demonstrated a 10% to 14% increased risk of stroke among patients with hypothyroid AIT after extensive consideration of confounding and potential biases. Given the relatively high prevalence of AIT and the morbidity and mortality associated with stroke, even small increases in stroke risk might be of high clinical relevance. The results of our study highlight the potential for regular screening for cardiovascular risk factors and preventive treatment (e.g., with statins) in patients with AIT.

## Supplementary Material

Data Supplement

## GLOSSARY

AF: atrial fibrillation
AIT: autoimmune thyroiditis
BMI: body mass index
CHD: coronary heart disease
CHF: congestive heart failure
CI: confidence interval
RR: rate ratio
THIN: The Health Improvement Network
