# Supplementary material for: Autoimmune thyroiditis as a risk factor for stroke: A historical cohort study
Source: Neurology. 2014 May 6;82(18):1643–52. doi: 10.1212/WNL.0000000000000377 (PMC4013815; doi:10.1212/WNL.0000000000000377)
Supplement: Data Supplement [file supp_82_18_1643__index.html]

Data Supplement 

# Autoimmune thyroiditis as a risk factor for stroke

## Data Supplement

One appendix and two tables; three Microsoft Word files.

**Neurology® data supplements are not copyedited before publication. Published editorials and translations have been copyedited.  
 © 2014 American Academy of Neurology.  
  
 Files in this Data Supplement:**

- Appendix e-1 - Microsoft Word file
- Table e-1 - Microsoft Word file
- Table e-2 - Microsoft Word file
