# Supplementary material for: Autoimmune thyroiditis as a risk factor for stroke: A historical cohort study
Source: Neurology. 2014 May 6;82(18):1643–52. doi: 10.1212/WNL.0000000000000377 (PMC4013815; doi:10.1212/WNL.0000000000000377)
Supplement: Data Supplement [file supp_WNL.0000000000000377_Appendix_e-1.docx]

**Appendix e-1:** Read codelists for exposure and outcome variables

## a) Read codelist for AIT

| READ code | Description |
| --- | --- |
| C045.00 | Acquired atrophy of thyroid |
| C04..00 | Acquired hypothyroidism |
| C046.00 | Autoimmune myxoedema |
| C052.11 | Autoimmune thyroiditis |
| F11x500 | Cerebral degeneration due to myxoedema |
| C053.00 | Chronic fibrous thyroiditis |
| C052.00 | Chronic lymphocytic thyroiditis |
| C05y400 | Chronic thyroiditis with transient thyrotoxicosis |
| C052.12 | Hashimoto's disease |
| C04z.13 | Hypothyroid goitre, acquired |
| C04..13 | Hypothyroidism |
| C04z.00 | Hypothyroidism NOS |
| 9Oj..00 | Hypothyroidism monitoring administration |
| 9Oj0.00 | Hypothyroidism monitoring first letter |
| 9Oj1.00 | Hypothyroidism monitoring second letter |
| 9Oj2.00 | Hypothyroidism monitoring third letter |
| 9Oj3.00 | Hypothyroidism monitoring verbal invite |
| F381400 | Myasthenic syndrome due to hypothyroidism |
| F395300 | Myopathy due to myxoedema |
| C04..11 | Myxoedema |
| C04z100 | Myxoedema coma |
| C04y.00 | Other acquired hypothyroidism |
| C05y.00 | Other and unspecified chronic thyroiditis |
| 4424200 | Serum T3 level low |
| C051.00 | Subacute thyroiditis |
| C06y100 | Thyroid atrophy |
| C04..12 | Thyroid deficiency |
| C04z.12 | Thyroid insufficiency |
| C05..00 | Thyroiditis |
| C05z.00 | Thyroiditis NOS |
| 66B9.11 | Thyroxine Rx started |
| 66BA.11 | Thyroxine Rx stopped |

b) Read codelist for stroke

| Read code | Description |
| --- | --- |
| G61..00 | Intracerebral haemorrhage |
| G61..11 | CVA - cerebrovascular accid due to intracerebral haemorrhage |
| G61..12 | Stroke due to intracerebral haemorrhage |
| G610.00 | Cortical haemorrhage |
| G611.00 | Internal capsule haemorrhage |
| G612.00 | Basal nucleus haemorrhage |
| G613.00 | Cerebellar haemorrhage |
| G614.00 | Pontine haemorrhage |
| G615.00 | Bulbar haemorrhage |
| G616.00 | External capsule haemorrhage |
| G617.00 | Intracerebral haemorrhage, intraventricular |
| G618.00 | Intracerebral haemorrhage, multiple localized |
| G61X.00 | Intracerebral haemorrhage in hemisphere, unspecified |
| G61X000 | Left sided intracerebral haemorrhage, unspecified |
| G61X100 | Right sided intracerebral haemorrhage, unspecified |
| G61z.00 | Intracerebral haemorrhage NOS |
| G62..00 | Other and unspecified intracranial haemorrhage |
| G62z.00 | Intracranial haemorrhage NOS |
| G630.00 | Basilar artery occlusion |
| G631.00 | CAROTID ARTERY OCCLUSION |
| G631.12 | Thrombosis, carotid artery |
| G632.00 | VERTEBRAL ARTERY OCCLUSION |
| G63y000 | Cerebral infarct due to thrombosis of precerebral arteries |
| G63y100 | Cerebral infarction due to embolism of precerebral arteries |
| G64..00 | Cerebral arterial occlusion |
| G64..11 | CVA - cerebral artery occlusion |
| G64..12 | Infarction - cerebral |
| G64..13 | Stroke due to cerebral arterial occlusion |
| G640.00 | Cerebral thrombosis |
| G640000 | Cerebral infarction due to thrombosis of cerebral arteries |
| G641.00 | Cerebral embolism |
| G641.11 | Cerebral embolus |
| G641000 | Cerebral infarction due to embolism of cerebral arteries |
| G64z.00 | Cerebral infarction NOS |
| G64z.11 | Brainstem infarction NOS |
| G64z.12 | Cerebellar infarction |
| G64z000 | Brainstem infarction |
| G64z200 | Left sided cerebral infarction |
| G64z300 | Right sided cerebral infarction |
| G64z400 | Infarction of basal ganglia |
| G66..00 | Stroke and cerebrovascular accident unspecified |
| G66..11 | CVA unspecified |
| G66..12 | Stroke unspecified |
| G66..13 | CVA - Cerebrovascular accident unspecified |
| G667.00 | Left sided CVA |
| G668.00 | Right sided CVA |
| G671000 | Acute cerebrovascular insufficiency NOS |
| G677000 | Occlusion and stenosis of middle cerebral artery |
| G677100 | Occlusion and stenosis of anterior cerebral artery |
| G677200 | Occlusion and stenosis of posterior cerebral artery |
| G677300 | OCCLUSION AND STENOSIS OF CEREBELLAR ARTERIES |
| G677400 | Occlusion+stenosis of multiple and bilat cerebral arteries |
| G6W..00 | Cereb infarct due unsp occlus/stenos precerebr arteries |
| G6X..00 | Cerebrl infarctn due/unspcf occlusn or sten/cerebrl artrs |
| Gyu6200 | [X]Other intracerebral haemorrhage |
| Gyu6300 | [X]Cerebrl infarctn due/unspcf occlusn or sten/cerebrl artrs |
| Gyu6400 | [X]Other cerebral infarction |
| Gyu6500 | [X]OCCLUSION AND STENOSIS OF OTHER PRECEREBRAL ARTERIES |
| Gyu6600 | [X]Occlusion and stenosis of other cerebral arteries |
| Gyu6F00 | [X]Intracerebral haemorrhage in hemisphere, unspecified |
| Gyu6G00 | [X]Cereb infarct due unsp occlus/stenos precerebr arteries |

c) Read codelist for TIA

| **Read code** | **Description** |
| --- | --- |
| Fyu5500 | [X]Other transient cerebral ischaemic attacks+related syndroms |
| G65..00 | Transient cerebral ischaemia |
| G65..12 | Transient ischaemic attack |
| G65y.00 | Other transient cerebral ischaemia |
| G65z.00 | Transient cerebral ischaemia NOS |
| G65z100 | Intermittent cerebral ischaemia |
| G65zz00 | Transient cerebral ischaemia NOS |
