# Supplementary material for: Autoimmune thyroiditis as a risk factor for stroke: A historical cohort study
Source: Neurology. 2014 May 6;82(18):1643–52. doi: 10.1212/WNL.0000000000000377 (PMC4013815; doi:10.1212/WNL.0000000000000377)
Supplement: Data Supplement [file supp_WNL.0000000000000377_Table_e-1.docx]

**Table e-1:** Changes in covariates during follow-up by exposure status (in people without the respective covariate at baseline)

| *Variable* |  | Adjusted RR** | p-value* |
| --- | --- | --- | --- |
| *Hypertension* | No AIT  AIT | 1  1.02 (0.99-1.05) | 0.162 |
| *Diabetes* | No AIT  AIT | 1  1.28 (1.22-1.33) | <0.001 |
| *Hyperlipidemia* | No AIT  AIT | 1  1.32 (1.28-1.36) | <0.001 |
| *Atrial fibrillation* | No AIT  AIT | 1  1.01 (0.99-1.04) | 0.621 |
| *Coronary Heart Disease* | No AIT  AIT | 1  1.23 (1.18-1.29) | <0.001 |
| *Chronic Heart Failure* | No AIT  AIT | 1  1.15 (1.09-1.21) | <0.001 |

*using LRTs

**adjusted for current age and sex and allowing for clustering in practice
