# Supplementary material for: Autoimmune thyroiditis as a risk factor for stroke: A historical cohort study
Source: Neurology. 2014 May 6;82(18):1643–52. doi: 10.1212/WNL.0000000000000377 (PMC4013815; doi:10.1212/WNL.0000000000000377)
Supplement: Data Supplement [file supp_WNL.0000000000000377_Table_e-2.docx]

**Table e-2:** Literature review of studies investigating the association of hypothyroidism with stroke and TIA

| Country  (Study design) | Source population | Definition of AIT/  hypothyroidism (n=) | Comparison group (n=) | Definition of outcome | Number of strokes in exposed | Final model adjusted for | Rate Ratio (95%CI) |
| --- | --- | --- | --- | --- | --- | --- | --- |
| Scotland (2006)[^23^](#_ENREF_23)  (Historical cohort) | Population-based, linking different databases | Treated hypothyroidism at baseline  **n=7,904** | General population (Standardized Incidence Ratio) | Ischemic stroke, hemorrhagic stroke or TIA (ICD 9 430-438  ICD 10 I60-69), hospital database | 160 | Age, Sex, Diabetes, pre-existing vascular disease | 1.25 (1.07-1.46) |
| USA (2006)[^7^](#_ENREF_7)  (Historical cohort) | Cardiovascular health study Age ≥65 years | At baseline:  TSH ≥20mU/l (overt)  **n=49**  4.5mU/l <TSH<20mU/l (subclinical)  **n=472** | Euthyroid patients  **n=2,502** | Stroke or TIA | 5 | Age, sex, clinical CVD at baseline, AF at baseline, thyroid  medication use during follow-up, race, smoking status, diabetes, cholesterol, use of lipid-lowering medications, hypertension, BMI, CRP | 0.86 (0.42-1-76)  1.01 (0.79-1.29) |
| USA (2006)[^25^](#_ENREF_25)  (Historical cohort) | NHANES**  Age 25-74 | At baseline:  Free Thyroxine index  <3.5mU/l  **n=493** | Euthyroid patients  **n=4,742** | Ischemic or hemorrhagic stroke (ICD 9 431-434.9, 437-437.1) | 38  (33 ischemic) | Age, sex, ethnicity, systolic blood pressure, smoking, obesity, diabetes, cholesterol | 1.6 (1.0-2.6) |
| USA (2005)[^12^](#_ENREF_12)  (Historical cohort) | Health, ageing, body composition study, Age 70-79 | At baseline:  TSH >4.5 mIU/l  **n=338** | Euthyroid patients  **n=2,392** | Stroke or TIA (using Cardiovascular Health Study diagnostic algorithms); telephone, hospital records and death certificates | 22 | Age, sex, ethnicity, smoking, diabetes, prevalent cardiovascular disease, poor helath, blood pressure, total cholesterol, creatinine, education, income, use of thyroid hormone, use of ACE inhibitors | 1.37 (0.85-2.20) |
| Germany (2008)[^22^](#_ENREF_22)  (Cross-sectional) | Population based sample in former iodine-deficient area Age 20-79 | TSH >2.12 mIU/l  **n=63** | Euthyroid patients, **n=1,839** | Self-reported physician’s diagnoses (stroke) | 2 | age, sex, BMI, hypertension, diabetes, smoking, school education, statins, low-density lipoprotein cholesterol, plasma fibrinogen | “No evidence” |
| Turkey (2007)[^24^](#_ENREF_24)  (Case-control) | Hospital patients | TSH ≥4.94µIU/ml  **n=10** | Neurology outpatients | Clinical diagnosis, confirmed by MRI (stroke) | 249 | N.R.* | N.R.* (p<0.05) |
| USA (2006)^26^  (Case-control) | Hospital patients | Hypothyroidism | N.R.* | Ischemic stroke | N.R.* | N.R.* | “significant difference” |
| UK, Present study  (Historical cohort) | Population-based database | Hypothyroid AIT patients treated with thyroxine  **n=34,907** | **n=149,632** | Ischemic stroke, hemorrhagic stroke (ICD 9 and 10-based Read code list) | 711 | current age, sex, alcohol, smoking; hypertension, AF, hyperlipdemia, and BMI (all at baseline) | 1.10 (1.01-1.20) |

*Not reported

NHANES=National Health and Nutrition Examination Survey; CVD=cardiovascular disease; AF=atrial fibrillation; CRP=C-reactive protein; BMI=body-mass-index; TSH=thyroid-stimulating hormone
